# Association between tumour somatic mutations and venous thromboembolism in the 100,000 Genomes Project cancer cohort: a study protocol

**DOI:** 10.12688/wellcomeopenres.23156.2

**Published:** 2024-12-24

**Authors:** Naomi Cornish, Sarah K. Westbury, Matthew T. Warkentin, Chrissie Thirlwell, Andrew D. Mumford, Philip C. Haycock

**Affiliations:** 1 School of Cellular and Molecular Medicine, University of Bristol, Bristol, England, BS82BN, UK; 2 University of Bristol Medical School, Bristol, England, BS82BN, UK; 3 Department of Oncology, University of Calgary Cumming School of Medicine, Calgary, Alberta, Canada

**Keywords:** Cancer, venous thromboembolism, tumour, somatic, mutation, genomic

## Abstract

Venous thromboembolism (VTE) is a common cause of morbidity and mortality in patients with cancer. There is evidence that specific aberrations in tumour biology contribute to the pathophysiology of this condition. We plan to examine the association between tumour somatic mutations and VTE in an existing cohort of patients with cancer, who were enrolled to the flagship Genomics England 100,000 Genomes Project. Here, we outline an a-priori analysis plan to address this objective, including details on study cohort selection, exposure and outcome definitions, annotation of genetic variants and planned statistical analyses. We will assess the effect of 1) deleterious somatic DNA variants in each gene; 2) tumour mutational burden and 3) tumour mutational signatures on the rate of VTE (outcome) in a pan-cancer cohort. Sensitivity analyses will be performed to examine the robustness of any associations, including adjustment for potentially correlated co-variates: tumour type, stage and systemic anti-cancer therapy. We hope that results from this study may help to identify key genes which are implicated in the development of cancer associated thrombosis, which may shed light on related mechanistic pathways and/or provide data which can be integrated into genetic risk prediction models for these patients.

## Plain Language Summary

People with cancer have a significantly higher risk of developing a condition known as venous thromboembolism (a type of blood clot). This is a major cause of illness and death. Rates of venous thromboembolism vary between different types of cancer and the reasons for this are not well understood. The purpose of this study is to evaluate whether there is a link between acquired genetic variants (which occur within the tumour) and venous thromboembolism. This study will hopefully contribute to our understanding of the mechanisms which drive the development of cancer-associated venous thromboembolism and help future efforts to develop models for more accurately identifying people at highest risk of the condition.

## Introduction

Venous thromboembolism (VTE) is a frequent complication of malignancy and a leading cause of cancer-related death ^1^. Estimates of the absolute risk of VTE in cancer patients vary enormously, from approximately 0.6% to over 20%, depending on a myriad of incompletely-understood risk factors ^2^. These include patient, cancer-specific and treatment-related variables ^3^.

One of the most powerful predictive factors for cancer-associated VTE is the anatomical type of tumour ^4^. Many tumours express elevated levels of prothrombotic proteins, or directly interact with adhesion molecules on platelets and vascular endothelial cells ^3, 5^. This suggests that VTE may result from specific aberrations of tumour biology.

Several groups have previously explored the association between alterations within the tumour genome and VTE. The majority of these studies are relatively small and have focused on a handful of candidate genes (including KRAS, EGFR, ALK and IDH1), sometimes with conflicting results ^6, 7^.

The largest study in this field used the Memorial Sloan Kettering (MSK)-IMPACT platform to sequence target genes in 11,695 tumour samples ^8^. Their pan-cancer analysis was restricted to 53 known oncogenes or tumour-suppressor genes, and found that somatic mutations in 7 genes modulated the risk of VTE. A separate analysis in the same cohort found several of these genes were also implicated in the development of arterial thrombosis ^9^. Recently, another publication from the MSK group has reported that levels of circulating tumour DNA independently predict VTE risk in a dose-dependent relationship. Interestingly some gene level alterations (including KRAS, STK11 and KEAP1) were also shown to be associated with VTE in the discovery cohort (n = 4,141) ^10^.

There is scope to attempt to replicate these results in an independent cohort and to further explore associations between VTE and somatic mutations in genes which have not previously been analysed.

## Objective

We plan to conduct a pan-cancer analysis examining the association between somatic mutations across the tumour genome (exposure) and VTE (outcome). 

We intend to analyse the exposure variable in 3 alternative ways **1) a gene-centric approach**, where the effect of somatic mutations is considered separately for each gene; **2)** by assessing **tumour mutational burden;** and **3)** by assessing **tumour mutational signatures.** The effect of these variables on the rate of VTE will be assessed using statistical models described below.

## Study population

This analysis uses existing data from the 100,000 Genomes Project Cancer Programme, a Genomics England (GEL) initiative which recruited 17,241 participants with cancer and performed whole genome sequencing (WGS) on matched germline and somatic (tumour) genomes ^11^. Recruitment occurred between 2015 and 2019. Eligibility for the overall program covered patients aged from birth upwards, diagnosed with a solid organ or haematological malignancy. Previously treated patients presenting with cancer recurrence, progression or undergoing surgery following neoadjuvant chemotherapy were all included. Genomic data is linked to pseudo-anonymised longitudinal electronic health records from secondary care, including Hospital Episode Statistics (HES), the National Cancer Registration and Analysis Service (NCRAS), the Systemic Anti-Cancer Therapy (SACT) dataset, and mortality data from the Office for National Statistics (ONS).

From the 100,000 Genomes Project version 18 data-release (December 2023), we will select a cohort of participants who meet the following inclusion criteria:

1.   Ongoing valid study consent and prospectively collected tumour sample (exclude patients with stored samples which were collected prior to study recruitment opening)

2.   Paired somatic and germline WGS meeting the following quality control criteria: concordant phenotypic and karyotypic sex; read mapping quality > 30 across 210 Gb for tumour DNA and 85Gb for germline DNA; cross-sample contamination <3% for germline DNA (assessed by VerifyBamID [ https://github.com/statgen/verifyBamID]) and <5% for tumour DNA (assessed by ConPair [ https://github.com/nygenome/Conpair]) ^12^.

3.   Linked NCRAS record with a congruous cancer diagnosis to the tumour type received by GEL.

4.   Histology consistent with malignant cancer.

5.   No missing or discrepant information for critical covariates (including age, sex, genetically inferred ancestry, cancer type and diagnosis date).

6.   No prior history of VTE (patients with a documented VTE prior to study entry will be excluded).

## Primary outcome definition

The primary outcome of interest is the first occurrence of VTE. This is a composite phenotype which we will identify using ICD10 codes (version:2019) recorded in linked hospital episode statistics (including inpatient, outpatient and emergency department records). Death from any cause other than VTE will be recorded from linked ONS data.

The ICD10 codes used to define VTE have been extracted from Health Data Research UK coding algorithms for deep vein thrombosis (PH338) and pulmonary embolism (PH71) [ https://phenotypes.healthdatagateway.org] ^13, 14^. In addition to the codes listed in the original algorithms, we have included codes I808-9 and I828-9 which encompass VTE at other/unspecified sites ( Table 1). This adaptation aims to ensure that upper limb VTE diagnoses are also identified, as these events are more common in cancer patients ^15^.

**Table 1.** ICD10 version: 2019 codes for venous thromboembolism in linked electronic health records.

Code	Description
**I26**	Pulmonary embolism
**I636**	Cerebral infarction due to cerebral venous thrombosis, non-pyogenic
**I676**	Non-pyogenic thrombosis of intracranial venous system
**I801**	Phlebitis and thrombophlebitis of femoral vein
**I802**	Phlebitis and thrombophlebitis of other deep vessels of lower extremities
**I808**	Phlebitis and thrombophlebitis of other sites
**I809**	Phlebitis and thrombophlebitis of unspecified site
**I81**	Portal vein thrombosis
**I820**	Budd-Chiari syndrome
**I822**	Embolism and thrombosis of vena cava
**I823**	Embolism and thrombosis of renal vein
**I828**	Embolism and thrombosis of other specified veins
**I829**	Embolism and thrombosis of unspecified vein

In line with recent literature in this field ^16, 17^, we have not included code I800 (phlebitis and thrombophlebitis of superficial vessels of lower extremities) in the case definition. This diagnosis is unlikely to be recorded accurately in hospital records of study participants as it is generally managed in the community. Furthermore, since superficial vein thrombosis of the leg is primarily associated with varicose veins ^18^, including these patients as VTE cases may reduce the power of our analysis to detect the effects of tumour genomic aberrations on rates of VTE.

## Covariate assessment

Baseline covariates (including age and sex) will be obtained from participant data submitted at recruitment. Cancer-specific covariate information will be derived from linked NCRAS records: this includes date of cancer diagnosis, tumour site and histological type, date of chemotherapy or surgery and stage of cancer (recorded within 12months of study entry). NCRAS diagnoses are coded using ICD10 version 19 [ https://icd.who.int/browse10/2019/en#/II]. Where applicable, information on the specific anti-cancer drug regimen administered will be obtained from linked SACT data.

## Genetic data

For cancer participants enrolled to the 100,000 Genomes Project, tissue collection and DNA extraction was performed in local laboratories linked to the regional NHS Genomics Medicine Centres. Tumour DNA was obtained primarily from fresh-frozen histology specimens (and rarely from formalin-fixed paraffin-embedded samples). Germline DNA was obtained from blood or occasionally saliva. The protocols for sample collection and DNA extraction are described in the GEL Sample Handling Guidance (v.4.0) available from [ Document library | Genomics England]. 

Samples were prepared with an Illumina TruSeq PCR-free library preparation kit, providing sufficient DNA was available; otherwise PCR-based library preparation was used for a minority of samples. DNA sequencing was performed centrally on an Illumina HiSeq platform to an average coverage of 30x (germline DNA) and 100x (tumour DNA). Sequence data was processed using the Illumina North Star (version 2.6.53.23) pipeline with read alignment against the human reference genome GRCh38-Decoy+EBV using ISAAC (version iSAAC-03.16.02.19) ^12^.

### Somatic variant calls and interpretation

Detection of somatic single nucleotide variants (SNVs) and insertions/deletions < 50bp has been performed using Strelka4 (v2.4.7) [ https://github.com/Illumina/strelka]. Detection of large structural somatic variants (inversions, translocations) and insertions/deletions >50bp has been performed using Manta (v.0.28.0) [ https://github.com/Illumina/manta]. In addition to the default quality filters applied by these pipelines, we will exclude 1) variants with germline allele frequency >1% in the GEL cohort (as these may indicate unsubtracted germline SNVs); 2) variants with somatic allele frequency >5% in the GEL cohort (as these may indicate potential technical artefacts); 3) indels in regions of high sequencing noise (i.e proportion of low quality filtered base calls within 50bp of the variant exceeds 10%) ^12^.

Variants have been annotated against the canonical transcript using Cellbase ^19^ (integrating information from ENSEMBL (version 90) ^20^, COSMIC (version 86) ^21^ and ClinVar (October 2018 release) ^22^. We will classify variants as potentially deleterious if they fall into any of the following categories:

- Transcript ablation
- Splice acceptor / splice donor variant
- Stop gain / loss
- Start loss
- In-frame insertion / deletion
- Frameshift
- Missense variant predicted to be deleterious to protein function by in-silico prediction algorithms (e.g. FATHMM-MKL score ^23^)
- Listed in Clinvar [ https://ncbi.nlm.nik/gov/clinvar] as pathogenic/likely pathogenic

### Gene inclusion criteria for gene-centric analysis

1)   We will analyse all genes which are listed in either tier 1 or tier 2 of the Cancer Gene Census [ https://cancer.sanger.ac.uk] ^21^. Tier 1 includes known oncogenic and tumour suppressor genes, while tier 2 includes genes where there is emerging (but less extensive) evidence to implicate their role in cancer pathology. Since it is becoming increasingly routine to perform sequencing of these genes in newly-diagnosed cancer patients ^24^, identifying whether there are associations between these genes and VTE risk has potential clinical utility in the near-future.

2)   We will analyse all genes for which germline polymorphisms have been shown to be associated with VTE in large population genome-wide or exome-wide association studies ^25– 27^ as we hypothesise that somatic variation in these same genes may contribute to cancer associated VTE.

3)   Finally, since this is an exploratory analysis designed to identify new genes which have not previously been implicated in VTE, we will analyse any remaining genes for which at least 5% of the study cohort carry potentially deleterious somatic variants. This cut-off has been chosen based on power calculations indicating that in a sample of ~10,000 participants, with a VTE prevalence ~ 10%, there will be >80% power to detect an association between VTE and mutations which are present in >5% of the study cohort, assuming an effect size of 1.5 and type 1 error rate < 0.05 ^28^.

For each gene included in the analysis we will dichotomise patients into one of two categories:

1) **Gene mutated:** if the **participant carries one or more potentially deleterious somatic variants** in the gene **at a variant allele frequency (VAF) >=5% in the tumour sample**. VAF is calculated by dividing the number of variant reads by the total number of reads (variant + reference sequence) at that position ^12^. This VAF threshold is derived from literature which suggests that variants called at a lower VAF are frequently due to sequencing errors ^29^.

**2)** **Gene unmutated:** If the participant **does not have any somatic variants** in the gene, **has a variant which is not predicted to be deleterious or has a variant present with a VAF < 5%**.

### Global tumour mutational burden

In addition to the gene-centric approach described above, we will consider the association between global tumour mutational burden (calculated as the total number of small somatic variants and indels per Mb of coding sequence) and risk of VTE.

### Mutational signature analysis

We will also report the association between 30 specific mutational signatures [ https://cancer.sanger.ac.uk/cosmic/signatures_v2] and risk of VTE. The contribution of each mutational signature to the overall mutation burden has been computed by GEL using the R package nnls ^30^.

## Statistical analyses

### Primary analysis

Our primary objective is to identify whether there are potentially causal relationships between somatic mutations in the tumour of a person with cancer and VTE. We will therefore use Cox proportional hazards regression to assess the effect of **1) deleterious somatic DNA variants in each gene; 2) tumour mutational burden and 3) tumour mutational signatures** on the **rate (hazard) of VTE (outcome)** in the pan-cancer cohort. We have chosen this model over a competing risks analysis, based on consensus in the literature that cause-specific hazard models are more appropriate for research questions focused around etiology, rather than prediction which requires accurate estimates of absolute risk that account for competing risks of mortality ^31^.

Time under observation will begin from the first date that a participant’s tumour was sampled for sequencing (here-after referred to as study entry). For participants who have had multiple tumour samples submitted to GEL, only DNA samples submitted at initial study entry will be used for analysis. Follow-up time will be defined as the time from study entry until the diagnosis of VTE, death from any cause, the last date when electronic health records were uploaded to the GEL Research environment (July 2022), or administrative study termination after five years, whichever occurs first. Diagnosis of VTE will be considered as the event of interest while all other events will be considered as right censoring of the follow-up time.

**Figure 1.**
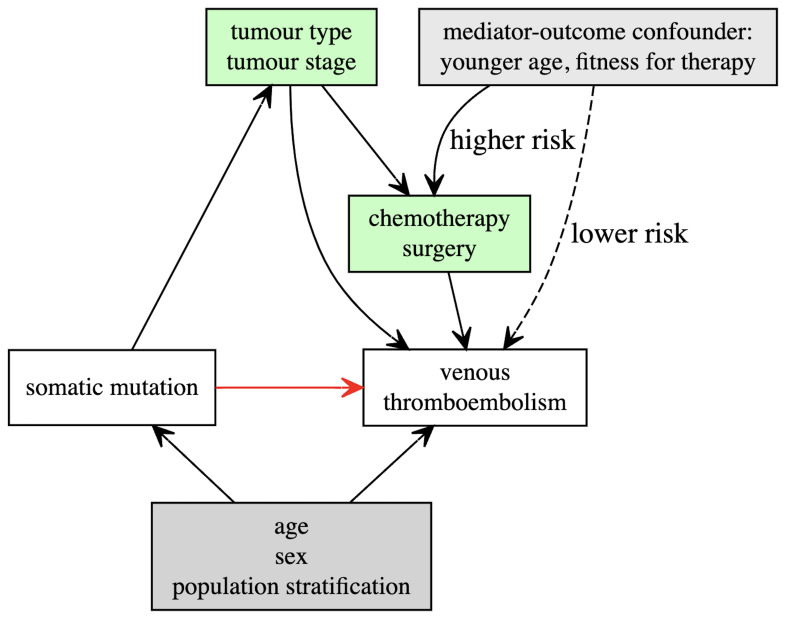
Directed acyclic graph illustrating hypothesised relationships between somatic mutations and venous thromboembolism, including potential confounders (grey) and mediators (green).

In the primary analysis, we will adjust Cox models for baseline patient covariates which we have identified as potential confounders including: age at study entry, sex and the top 4 genetic principal components ( Figure 1) **(minimally adjusted model)**.

To prevent convergence issues in the regression model caused by complete separation (due to zero / near-zero outcome events in an exposure group) ^32^, we will limit the analyses to genes/mutational signatures where at least 5 VTE events occur in each exposure category.

Results will be expressed in terms of the hazard ratio (HR) and associated 95% confidence interval (95% CI) for VTE associated with each gene and mutational signature. For tumour mutational burden, we will standardize the exposure variable and report the HR and 95% CI for VTE per standard deviation increase in mutation burden. Results will be interpreted in the context of false-discovery rate corrected p-values to account for multiple testing.

We will assess for violations of the proportional hazards assumption underlying our statistical models by incorporating time-dependent interaction terms to examine whether the relative association between somatic mutations and VTE changes over time, and by evaluating Schoenfeld residuals.

### Sensitivity analyses

***Sensitivity analysis 1:* Examine interactions between somatic mutations and other covariates which may mediate or moderate the association with VTE.** We hypothesise that somatic mutations arising within a tumour contribute directly to VTE (for example through increased tumour-expression of pro-thrombotic proteins). However, these same mutations may drive tumour growth or proxy aggressive histological subtypes of malignancy. This may lead to an indirect association with VTE via other mechanisms ( Figure 1). For example, large tumours may compress or invade vessels, contributing to venous stasis and subsequent thrombosis. Patients with advanced cancer are also more likely to experience complications such as infection or hospitalization, all of which have been associated with VTE ^3, 5^. Chemotherapy and surgery are also strongly associated with VTE risk. In the era of molecularly targeted therapies, selection of systemic anti-cancer drugs may be dictated by knowledge of the underlying somatic mutation profile ^33^. This may lead to associations between genetic variants and VTE which are mediated through drugs, rather than any direct biological phenomenon.

We are interested primarily in estimating the degree to which somatic mutations contribute to VTE through direct biological mechanisms. Therefore, for genes / gene signatures where there is possible evidence from the primary analysis for an association with VTE (nominal P < 0.05), we will conduct mediation analyses to explore the degree to which these associations are dependent on indirect pathways/mediating variables

We will compare estimates from the minimally adjusted (primary analysis) with estimates from Cox regressions where we include the following covariates (presumed mediators of the exposure-outcome relationship): 1) tumour type, grouped by anatomical site; 2) cancer stage and 3) SACT. SACT will be included as a time-dependent covariate. We will also group SACT regimens by type and include these drug groups as distinct covariates. Participants with missing information on any of the above covariates will be excluded from this sensitivity analysis.

It is also plausible that baseline germline thrombophilia polymorphisms modulate the impact of tumour genetic changes on rates of VTE. We will therefore examine the interaction between tumour somatic mutations in each gene and a 297-SNP germline polygenic risk score (derived from a GWAS by Klarin *et al.*) ^34^ on the rate of VTE. We have selected this score as, compared with other VTE polygenic risk scores, it demonstrated superior predictive performance for cancer-associated thrombosis when evaluated in the UK biobank cancer population ^16^.

***Sensitivity analysis 2: Competing risks analysis.*** For genes/mutational signatures where there is statistical evidence indicating a possible association with the rate of VTE in the primary analysis (nominal p <0.05), we will use Fine and Gray regressions to estimate the risk of VTE in the gene-mutated vs reference groups, treating death from any other cause as a competing risk. We will report the subdistribution hazard ratio (SHR) and associated 95% CI for VTE associated with the gene/mutational signature, as well as the estimated cumulative incidence for VTE over time ^31^.

***Sensitivity analysis 3: Exclude relapsed and previously treated participants.*** The GEL cancer cohort includes some pre-treated patients with relapsed or progressive disease as well as patients who have undergone neo-adjuvant chemotherapy prior to study entry. Prior chemotherapy may be a powerful risk factor for VTE ^5^. It may also influence the somatic mutation profile of a tumour through treatment-induced mutagenesis and selection of tumour sub-clones ^35^. Therefore, we will perform a sensitivity analysis which is limited to only newly diagnosed untreated cancer patients: excluding any patients who have received SACT prior to study entry or where the date between original cancer diagnosis and study entry is > 180 days.

To control for variability in time between cancer diagnosis and tissue biopsy (for somatic DNA sequencing), we will also perform a sensitivity analysis where the outcome is **time to VTE from cancer diagnosis**, with left truncation at the point of tumour sampling.

***Sensitivity analysis 4: Exclude patients with a documented indication for anticoagulation or anti-platelet therapy.*** Older patients with co-morbidities may be more likely to be prescribed anticoagulation for reasons other than VTE (for example, for stroke prevention in the context of atrial fibrillation) ^36^. This is a potential confounding variable and failing to include it as a co-variate may introduce bias. Prescription data are unfortunately not available for this cohort. However, we will perform a sensitivity analysis where we exclude patients who have a common medical indication for long-term anticoagulation or antiplatelet therapy documented in their hospital episode statistics prior to study entry: namely atrial fibrillation, acute coronary syndrome, ischaemic stroke/transient ischaemic attack or prosthetic heart valve replacement ^37, 38^. (Note patients with a prior history of VTE are excluded from the primary cohort).

***Sensitivity analysis 5: Ancestry stratified analysis.*** The cohort is predominantly comprised of participants of European genetic ancestry, which potentially limits the generalizability of any findings. Although power to detect ancestry-specific associations in non-Europeans will be low, we will assess the direction of effect of any gene associations with are identified from the primary analysis in an ancestry-stratified sensitivity analysis.

## Discussion

In this protocol, we plan to examine the impact of tumour somatic mutations on rates of VTE. We will attempt to adjust for factors which may be correlated with both tumour genetics and VTE, including age, sex, tumour site and stage of cancer. However, there will still be residual and unmeasured confounding which may bias our results. For example, obesity is a risk factor both for the development of some cancers and for VTE ^39^. Unfortunately, weight and height has been recorded for fewer than 5% of participants in the 100,000 Genomes Project cancer programme. Therefore, it is not possible to include body mass index as a covariate. Similarly, haematological laboratory parameters (including haemoglobin, leucocyte count and platelet count) which have previously been shown to be relevant in the clinical prediction of cancer-associated thrombosis ^40^, are not available for this dataset.

The longitudinal health record data for participants in our cohort is obtained through linkage with pseudo-anonymised hospital-episode statistics (coded using ICD10 diagnoses). Unfortunately, this does not include full imaging reports or free-text hospital notes. It is therefore not possible to directly validate the specificity and sensitivity of ICD10 codes for VTE in the context of this study. We note recent work by Overvad *et al.* ^17^ showing that identification of cancer-associated VTE through ICD10 codes recorded in electronic health registry data was reasonably specific (positive predictive value >86%) and sensitive (~74% of oncology patients with venous thromboembolism had a relevant ICD10 code documented). We acknowledge that deriving diagnoses from administrative hospital statistics will inevitably lead to some degree of outcome misclassification, particularly given that no primary care data is currently available for participants, meaning VTE diagnoses which are only recorded in GP records will be missed. We anticipate this will result in an under-estimation of the true VTE incidence, which could potentially bias effect estimates towards the null (although bias in the other direction is also plausible) ^41^.

Risk of VTE is dynamic and likely to change over the course of a patient’s cancer illness. It may also be moderated by events such as hospital admissions and the administration of short or medium-term primary thromboprophylaxis. As we do not have prescription data available, we cannot specifically explore the impact of these factors. However, we will examine our data to ascertain whether the relative effect of somatic mutations on VTE varies over time and interpret our results in light of these considerations.

Despite these limitations, we believe this large pan-cancer cohort, with good quality paired tumour and germline whole genome sequence data and individual participant linkage to longitudinal health records, provides a unique opportunity to study the contribution of tumour genomic abnormalities to VTE pathophysiology. It is outside the scope of this analysis to attempt to develop or validate a comprehensive risk-prediction model. However, we hope that any gene associations which we identify can be carried forward into future (ideally prospectively-designed) studies to evaluate the value of adding tumour genome sequence data to existing risk-prediction algorithms for cancer associated thrombosis.

## Study status

Prior to finalising this protocol, NC explored the GEL research environment platform to develop familiarity with the data structure and the available files for analysis, in order to inform judgements on the feasibility of the planned study.

To ascertain whether there was likely to be adequate power to perform the analyses described above (page 7), we also generated descriptive statistics on the overall cancer cohort including

- Proportion of the sample with missing covariate data
- Proportion of the population experiencing the outcome of interest (VTE)

No prior analyses examining the associations between somatic mutations and VTE have been performed.

Future amendments to this protocol will be clearly documented and justified in subsequent reports. We plan to submit findings from this study for publication once analyses are complete.

## Ethics and consent

This study has been approved by the Genomics England research network, and access to the data will adhere to Genomics England research governance agreements. The 100,000 Genomes Project was approved by the NHS Health Research Authority East of England - Cambridge South Research Ethics Committee (REC ref: 14/EE/1112, 20 ^th^ February 2015). Participants were recruited from across 13 NHS Genomic Medicine Centres and all participants agreed to participate in the 100,000 Genomes Project and provided informed written consent.

## Data Availability

The de-identified patient data which will be used for this analysis can be accessed via the Genomics England Research Environment subject to a collaborative agreement that adheres to patient-led governance. For more information about accessing the data, contact research-network@genomicsengland.co.uk or access the relevant information on the Genomics England website: https://www.genomicsengland.co.uk/research. **University of Bristol data repository** [ https://data.bris.ac.uk/data/] : STROBE checklist for ‘Association between tumour somatic mutations and venous thromboembolism in the 100,000 Genomes Project cancer cohort: a study protocol’. https://doi.org/10.5523/bris.1pmmcgyqaij8n27j94i2rgd4j9 ^42^. Data are available under the terms of the Creative Commons Attribution 4.0 International license (CC-BY 4.0).

## Software availability

Analyses will be carried out in R version 4.2.1 using standard open source software packages available from [ https://cran.r-project.org] including survival, cmprsk and riskRegression ^43– 45^.

All analysis scripts will be posted publicly on github [ https://github.com/NaomiC-0] following an airlock approval from the Genomics England data protection committee.
