# Supplementary material for: Association between tumour somatic mutations and venous thromboembolism in the 100,000 Genomes Project cancer cohort: a study protocol
Source: Wellcome Open Res. 2024 Dec 24;9:640. Originally published 2024 Nov 4. [Version 2] doi: 10.12688/wellcomeopenres.23156.2 (PMC11809147; doi:10.12688/wellcomeopenres.23156.2)
Supplement: Peer review history

# Reviewer report: Reviewer response for version 2

**DOI:** 10.21956/wellcomeopenres.25990.r116319

**Published:** 2024-12-30

**Authors:** Yan Xu

**Affiliations:** 1 University of Ottawa and The Ottawa Hospital Box 201a, Ottawa, Canada

The authors have adequately addressed the queries raised in my original review, and added additional analyses as applicable (e.g., germline predispositions to VTE). I have no further comments and wish the authors the best in their analysis which I anticipate with great interest.

Is the study design appropriate for the research question?

Yes

Is the rationale for, and objectives of, the study clearly described?

Yes

Are sufficient details of the methods provided to allow replication by others?

Yes

Are the datasets clearly presented in a useable and accessible format?

Yes

Reviewer Expertise:

Venous thromboembolism

I confirm that I have read this submission and believe that I have an appropriate level of expertise to confirm that it is of an acceptable scientific standard.

# Reviewer report: Reviewer response for version 2

**DOI:** 10.21956/wellcomeopenres.25990.r116320

**Published:** 2024-12-27

**Authors:** Benilde Cosmi

**Affiliations:** 1 University of Bologna, Bologna, Italy

The authors have adequately addressed previously raised concerns.

Is the study design appropriate for the research question?

Yes

Is the rationale for, and objectives of, the study clearly described?

Yes

Are sufficient details of the methods provided to allow replication by others?

Yes

Are the datasets clearly presented in a useable and accessible format?

Yes

Reviewer Expertise:

venous thromboembolism , heparin, vitamin K antagonists, direct oral anticoagulant

I confirm that I have read this submission and believe that I have an appropriate level of expertise to confirm that it is of an acceptable scientific standard.

# Reviewer report: Reviewer response for version 1

**DOI:** 10.21956/wellcomeopenres.25500.r110118

**Published:** 2024-12-05

**Authors:** Benilde Cosmi

**Affiliations:** 1 University of Bologna, Bologna, Italy

The authors plan to perform a whole genome sequencing  (WGS) in patients with cancer from 100,000 Genomes Project Cancer Programme, a Genomics England (GEL) initiative to assess the association between somatic mutations across the tumour genome (exposure) and venous thromboembolism (VTE ) (outcome).

**Some issues should be addressed as indicated below:**

  1- Outcomes: the diagnosis of DVT in any site and/or PE is based on ICD-10  as reported in table 1. However DVT of the upper limb is not considered but it is quite common in cancer patients ( mostly due to central venous catheters). Superficial vein thrombosis of the lower limbs was also excluded. The authors should justify these exclusion.

 2- In addition ICD-10 codes do not guarantee that VTE diagnosis was objectively documented.

 3- Sensitivity analysis 4: Cancer patients can also receive thromboprophylaxis in case of hospitalization for acute illness or surgery, and not only long term anticoagulation. In fact the risk of VTE is dynamic and it can change during the  clinical course of cancer. This may also impact on the risk of VTE.

Is the study design appropriate for the research question?

Yes

Is the rationale for, and objectives of, the study clearly described?

Yes

Are sufficient details of the methods provided to allow replication by others?

Yes

Are the datasets clearly presented in a useable and accessible format?

Yes

Reviewer Expertise:

venous thromboembolism , heparin, vitamin K antagonists, direct oral anticoagulant

I confirm that I have read this submission and believe that I have an appropriate level of expertise to confirm that it is of an acceptable scientific standard, however I have significant reservations, as outlined above.

# Response

**Published:** 2024-12-19

**Authors:** Naomi Cornish

**Affiliations:** University of Bristol School of Cellular and Molecular Medicine, Bristol, England, UK

Many thanks for taking the time to review this study protocol. We appreciate your valuable insights regarding the potential limitations of this analysis and where possible have incorporated your feedback to improve the revised manuscript. Our response to your comments is below:  

 1. The ICD10 codes which we propose using to identify venous thromboembolism from electronic health record data are derived from a previously published algorithm by Kuan *et al* which has been curated by a panel of clinicians. ^[1]^ We deliberately chose a standardised algorithm for venous thromboembolism in an effort to ensure that our outcome definition is easily reproducible. However the algorithm was not developed specifically for cancer-associated thrombosis, and, as you highlight, does not include upper limb deep vein thrombosis (DVT), which occurs more frequently in people with cancer. ^[2]^ Therefore, we have expanded the diagnostic algorithm to include codes I808-9 and I828-9 which encompass thromboembolism of ‘other’ or ‘unspecified’ veins (Table 1 of manuscript). Specific codes for upper extremity DVT (I826), axillary vein thrombosis (I82A), subclavian vein thrombosis (I82B) and internal jugular thrombosis (I82C) do not appear in the data. This is due to the fact that the health records are coded using WHO ICD10 version 19 ^[3]^, which lacks some of the more granular codes that appear in ICD10-CM (curated by the National Centre for Health Statistics, US). ^[4]^ In line with recent literature in this field ^[5,6]^ we have not included ICD10 code I800 (“Phlebitis and thrombophlebitis of superficial vessels of lower extremities”) in the case definition. This diagnosis is unlikely to be recorded accurately in hospital records of study participants as it is generally managed in the community. Furthermore, although superficial vein thrombosis undoubtedly shares risk factors with deep venous thrombosis, it may have distinct aetiology in this context, given that it is primarily associated with the presence of varicose veins and lower limb venous insufficiency. ^[7]^ Including these patients as VTE cases may reduce the power of our analysis to detect the direct effects of tumour genomic aberrations on thrombosis development. We have clarified our justification for this in the manuscript (primary outcome definition).  

 2. Unfortunately, the linked electronic health records for participants in this cohort do not include imaging reports or free-text hospital notes. It is therefore not possible to directly validate the specificity and sensitivity of ICD10 codes for venous thromboembolism in the context of this study.  We note recent work by Overvad *et al* ^[6]^ showing that identification of incident cancer-associated venous thromboembolism through ICD10 codes recorded in electronic health registry data was reasonably specific (positive predictive value >86%) and sensitive (~74% of oncology patients with venous thromboembolism had a relevant ICD10 code documented). We acknowledge that using administrative data to identify our primary outcome will inevitably lead to the misclassification of some participants. However, we would anticipate the impact of this would be more likely to bias effect estimates towards the null, by diluting the ‘true’ case vs control groups and thereby reducing the power to detect casual associations (rather than introducing false positive associations, although this is also possible). ^[8]^ We have acknowledged these limitations in the manuscript (discussion section).

   3. We agree that the risk of VTE is dynamic and likely to change over the course of a patient’s cancer illness. As we do not have prescription data available, we cannot explore the impact of short or medium-term anticoagulation prescriptions (for example in the context of hospital admissions) on the rate of venous thromboembolism. We have expanded on this limitation in the discussion section. We will examine whether the effect of somatic mutations on venous thromboembolism changes over time. This will include assessing the data for violations of the proportional hazards assumption underlying our statistical models, by incorporating time-dependent interaction terms and evaluating Schoenfeld residuals. We have added this clarification to the statistical analysis plan.  

**References:**

 1. Kuan V, Denaxas S, Gonzalez-Izquierdo A, Direk K, Bhatti O, Husain S, et al. A chronological map of 308 physical and mental health conditions from 4 million individuals in the English National Health Service. *Lancet Digit Health* 2019;1(2):e63–77.

 2. Alikhan R, Gomez K, Maraveyas A, Noble S, Young A, Thomas M, et al. Cancer-associated venous thrombosis in adults (second edition): A British Society for Haematology Guideline. *Br J Haematol *2024;

 3. World Health Organization. International statistical classification of diseases and related health problems (11th ed.) [Internet]. 2019 [cited 2024 Jul 31];Available from: https://icd.who.int/browse10/2019/en#/I80-I89.

 4. CDC. ICD-10-CM [Internet]. Classification of Diseases, Functioning, and Disability2024 [cited 2024 Dec 12];Available from: https://www.cdc.gov/nchs/icd/icd-10-cm/index.html.

 5. Guman NAM, Mulder FI, Ferwerda B, Zwinderman AH, Kamphuisen PW, Büller HR, et al. Polygenic risk scores for prediction of cancer-associated venous thromboembolism in the UK Biobank cohort study. *J Thromb Haemost *2023;S1538-7836(23)00571-8.

 6. Overvad TF, Severinsen MT, Johnsen SP, Madsen SS, Kannik K, Stenfeldt LG, et al. Positive predictive value and sensitivity of cancer-associated venous thromboembolism diagnoses in the Danish National Patient Register. T *hrombosis Research *2024;241:109074.

 7. Cosmi B. Management of superficial vein thrombosis. *Journal of Thrombosis and Haemostasis *2015;13(7):1175–83. 8. Bakoyannis G, Yiannoutsos CT. Impact of and Correction for Outcome Misclassification in Cumulative Incidence Estimation. *PLOS ONE *2015;10(9):e0137454.

# Reviewer report: Reviewer response for version 1

**DOI:** 10.21956/wellcomeopenres.25500.r113413

**Published:** 2024-11-28

**Authors:** Yan Xu

**Affiliations:** 1 University of Ottawa and The Ottawa Hospital Box 201a, Ottawa, Canada

In this protocol, Dr. Cornish and colleagues seek to establish an association between somatic mutation in tumoral environment and risk of venous thromboembolism through use of UK’s 100,000 Genomes Project. In addition to evaluating an association between tumoural somatic mutations and risk of VTE, the authors plan to adjust for several confounding variables (tumour type, stage, use of anti-cancer therapy) and perform sensitivity analyses to delineate the contribution from somatic mutations in context of other factors that contribute to increased risk of VTE.

 The authors have presented a clear proposal with important impact on care of patients living with cancer. As anti-cancer therapies evolve and our understanding of VTE risks among patients with cancer are refined, there is a need for a precision medicine approach to improve our primary VTE prevention methods.

**Major comments:**

1. The authors propose use of tumour type, stage and systemic anti-cancer therapy as mediators and age at study entry, sex and the top 4 genetic principal components as confounders. In addition, the authors are encouraged to consider components of other established risk scores for VTE prediction in cancer patients (e.g., Khorana score components) such as anemia, thrombocytosis and leukocytosis as mediators, as they likely reflect the underlying inflammatory state of malignancy and are well-established risk factors for VTE in cancer patients. Furthermore, BMI should be considered a confounder given the association between elevated BMI and development of both selected malignancies (e.g., post-menopausal breast, colorectal) and VTE. If these variables are not available, these should be acknowledged as limitations.
2. The authors should consider patient-related germline risk factors for cancer-associated VTE (e.g, polygenic risk score for cancer-associated VTE has been evaluated by Guman et al, JTH 2023). While one presumes that germline risk factors should not correlate with somatic mutations in the tumoural environment, they may modulate the risk of observed VTE if those with germline predispositions (e.g., factor V Leiden) have higher tendencies to receive thromboprophylaxis.
3. Impact of ancestry on somatic variants should be considered as there seems to be fewer Domain 1 variants and those from all domains combined ( Diversity and genetic ancestry effects in the… | Genomics England). Performance of specific somatic mutations on VTE should be evaluated in ancestral groups to ensure generalizability, rather than simply classifying PCA-defined ancestry as a mediator in the analysis.
4. Performance of ICD-10 codes (sensitivity, specificity, PPV, NPV) are not clear and would warrant validation.

Is the study design appropriate for the research question?

Yes

Is the rationale for, and objectives of, the study clearly described?

Yes

Are sufficient details of the methods provided to allow replication by others?

Yes

Are the datasets clearly presented in a useable and accessible format?

Yes

Reviewer Expertise:

Venous thromboembolism

I confirm that I have read this submission and believe that I have an appropriate level of expertise to confirm that it is of an acceptable scientific standard, however I have significant reservations, as outlined above.

## References

[1] : Polygenic risk scores for prediction of cancer-associated venous thromboembolism in the UK Biobank cohort study. *J Thromb Haemost* .2023;21(11) : 10.1016/j.jtha.2023.07.009 3175-3183 10.1016/j.jtha.2023.07.009 37481074

[2] Diversity and genetic ancestry effects in the Cancer Cohort of the 100,000 Genomes Project. *Genomics England* . Reference source

# Response

**Published:** 2024-12-19

**Authors:** Naomi Cornish

**Affiliations:** University of Bristol School of Cellular and Molecular Medicine, Bristol, England, UK

Thank you for taking the time to review our study protocol.  We are very grateful for your expertise and the effort you have taken to provide detailed feedback. We have responded to each of your comments below and, where possible, we have adapted our analysis plan according to your suggestions (revision uploaded).  

 1. Obesity is a potentially confounding variable which may influence the development of both cancer and venous thromboembolism. ^[1]^ Unfortunately, weight and height has been recorded for fewer than 5% of participants in the 100,000 Genomes Project cancer programme. Therefore, it is not possible to include body mass index as a covariate. Similarly, haematological laboratory parameters (including haemoglobin, leucocyte count and platelet count) are not available for this dataset. Although these variables have undoubtedly been shown to be relevant in the clinical prediction of cancer-associated thrombosis, ^[2]^ our primary aim in this analysis is specifically to examine the contribution of tumour genomic abnormalities to the aetiology of venous thromboembolism, rather than to develop or validate a comprehensive risk-prediction model. We also note that prospective studies have shown that clinical utility of the Khorana score is relatively low for several cancer sites. ^[3,4]^ Although it is outside the scope of this particular analysis, we hope that any gene associations which we identify can be carried forward into future studies to evaluate the value of adding tumour genome sequence data to existing risk-prediction algorithms. We have acknowledged these limitations in the updated manuscript (discussion section).  

 2. Recent UK guidelines do not currently suggest routine primary thromboprophylaxis for cancer patients who carry common germline thrombophilia variants (including Factor V Leiden). ^[5,6]^ However, we agree that germline predisposition to venous thromboembolism may well interact with tumour somatic mutations to modulate the risk of cancer-associated thrombosis. We therefore plan to examine whether a previously validated germline polygenic risk score ^[7]^ further affects the rate of venous thromboembolism in the context of different tumour somatic gene mutations (sensitivity analysis 1).

 3. We recognise that population stratification may potentially confound observed associations between somatic mutations and venous thromboembolism. We attempt to mitigate this by including genetic principal components as covariates in the proposed statistical models. However, as you highlight, associations identified from a predominantly European study cohort may not be generalisable to other diverse populations. Therefore, we have updated the protocol to include an ancestry-stratified analysis (sensitivity analysis 5), while acknowledging that our power to detect ancestry-specific associations will be limited, given the relatively low numbers of non-European ancestry participants. ^[8]^  

 4. The longitudinal health record data for participants in our cohort is obtained through linkage of pseudo-anonymised hospital-episode statistics (coded using ICD10 diagnoses) from NHS England. Unfortunately, this does not include full imaging reports or free-text hospital notes. It is therefore not possible to directly validate the specificity and sensitivity of ICD10 codes for venous thromboembolism in the context of this study.  We note recent work by Overvad *et al* ^[9]^ showing that identification of incident cancer-associated venous thromboembolism through ICD10 codes recorded in electronic health registry data was reasonably specific (positive predictive value >86%) and sensitive (~74% of oncology patients with venous thromboembolism had a relevant ICD10 code documented). We acknowledge that using administrative data to identify our primary outcome will inevitably lead to the misclassification of some participants. However, we would anticipate the impact of this would be more likely to bias effect estimates towards the null, by diluting the ‘true’ case vs control groups and thereby reducing the power to detect casual associations (rather than introducing false positive associations, although this is also possible). ^[10]^ We have acknowledged these limitations in the manuscript (discussion section).  

**References:**

 1. Larsson SC, Burgess S. Causal role of high body mass index in multiple chronic diseases: a systematic review and meta-analysis of Mendelian randomization studies. *BMC Medicine* 2021;19(1):320.

 2. Khorana AA, Kuderer NM, Culakova E, Lyman GH, Francis CW. Development and validation of a predictive model for chemotherapy-associated thrombosis. *Blood*. 2008;111(10):4902–7.

 3. Mulder FI, Candeloro M, Kamphuisen PW, Di Nisio M, Bossuyt PM, Guman N, et al. The Khorana score for prediction of venous thromboembolism in cancer patients: a systematic review and meta-analysis. *Haematologica* 2019;104(6):1277–87.

 4. Overvad TF, Ording AG, Nielsen PB, Skjøth F, Albertsen IE, Noble S, et al. Validation of the Khorana score for predicting venous thromboembolism in 40 218 patients with cancer initiating chemotherapy. *Blood Adv* 2022;6(10):2967–76.

 5. Arachchillage DJ, Mackillop L, Chandratheva A, Motawani J, MacCallum P, Laffan M. Thrombophilia testing: A British Society for Haematology guideline. *Br J Haematol  *2022;198(3):443–58.

 6. Alikhan R, Gomez K, Maraveyas A, Noble S, Young A, Thomas M, et al. Cancer-associated venous thrombosis in adults (second edition): A British Society for Haematology Guideline. *Br J Haematol *2024;

 7. Guman NAM, Mulder FI, Ferwerda B, Zwinderman AH, Kamphuisen PW, Büller HR, et al. Polygenic risk scores for prediction of cancer-associated venous thromboembolism in the UK Biobank cohort study. *J Thromb Haemost *2023;S1538-7836(23)00571-8.

 8. Nguyen BT, Kuchenbaecker K, Silver M, Tallman S, Cho Y, Sosinsky A, et al. Diversity and genetic ancestry effects in the Cancer Cohort of the 100,000 Genomes Project [Internet]. Genomics England2023 [cited 2024 Dec 11];Available from: https://www.genomicsengland.co.uk/blog/diversity-and-genetic-ancestry-effects-in-the-cancer-cohort-of-the-100-000-genomes-project

 9. Overvad TF, Severinsen MT, Johnsen SP, Madsen SS, Kannik K, Stenfeldt LG, et al. Positive predictive value and sensitivity of cancer-associated venous thromboembolism diagnoses in the Danish National Patient Register. *Thrombosis Research *2024;241:109074.

 10. Bakoyannis G, Yiannoutsos CT. Impact of and Correction for Outcome Misclassification in Cumulative Incidence Estimation. *PLOS ONE *2015;10(9):e0137454.
